# Kinetics of N_2_ Release from Diazo Compounds: A Combined Machine Learning-Density Functional Theory Study

**DOI:** 10.1021/acsomega.3c07367

**Published:** 2023-12-15

**Authors:** Kaveh Farshadfar, Arsalan Hashemi, Reza Khakpour, Kari Laasonen

**Affiliations:** Department of Chemistry and Material Science, School of Chemical Engineering, Aalto University, 02150 Espoo, Finland

## Abstract

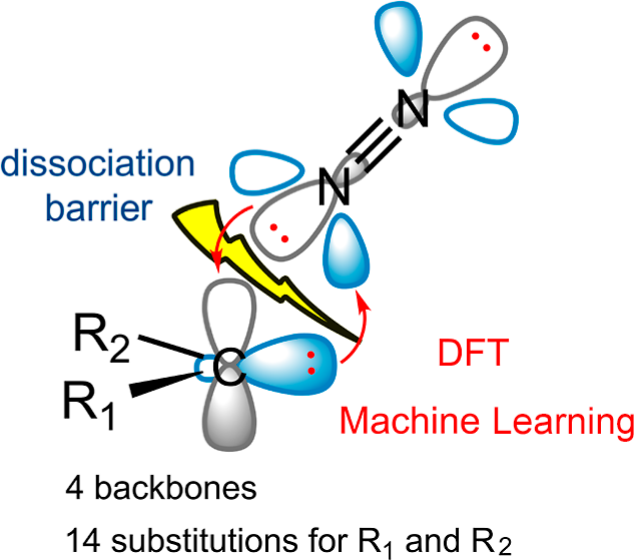

Diazo compounds are commonly employed as carbene precursors in carbene transfer reactions during a variety of functionalization procedures. Release of N_2_ gas from diazo compounds may lead to carbene formation, and the ease of this process is highly dependent on the characteristics of the substituents located in the vicinity of the diazo moiety. A quantum mechanical density functional theory assisted by machine learning was used to investigate the relationship between the chemical features of diazo compounds and the activation energy required for N_2_ elimination. Our results suggest that diazo molecules, possessing a higher positive partial charge on the carbene carbon and more negative charge on the terminal nitrogen, encounter a lower energy barrier. A more positive C charge decreases the π-donor ability of the carbene lone pair to the π* orbital of N_2_, while the more negative N charge is a result of a weak interaction between N_2_ lone pair and vacant p orbital of the carbene. The findings of this study can pave the way for molecular engineering for the purpose of carbene generation, which serves as a crucial intermediate for many chemical transformations in synthetic chemistry.

## Introduction

Diazo compounds [*i*.*e*., (R_1_)(R_2_)CN_2_ where R_1_ and R_2_ indicate functional groups] are well-known carbene [*i*.*e*., (R_1_)(R_2_)C] precursors that have garnered significant attention in organic synthesis because of their unique electronic and structural properties.^1−10^ The generated carbenes from diazo compounds are highly reactive intermediates that participate in a variety of chemical processes, including XH insertion (where X = C, O, N, and S),^11−18^ cyclopropanation,^19−24^ and tandem cross-coupling.^25−28^

**Scheme 1:**
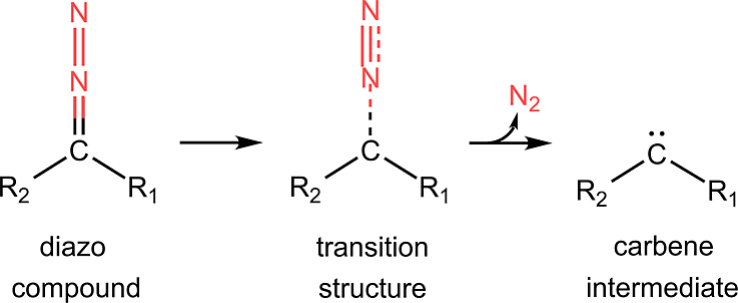
Schematic Representation of N_2_ Release from a Diazo Compound and Generation of Carbene

The N_2_ gas is released to the atmosphere from the diazo compound to carbene transformation (Scheme 1). Thus, if the activation energy barrier for N_2_ release is overcome, then the reaction becomes irreversible. In other words, regardless of whether the reaction is thermodynamically exothermic or endothermic, it is governed by kinetics. The kinetics of a reaction can be affected by molecular engineering, either at the backbone or at the function group level. There is, however, one important point to remember: not all the reactions lead to stable carbene intermediates because of intramolecular rearrangements.^29,30^

**Scheme 2:**
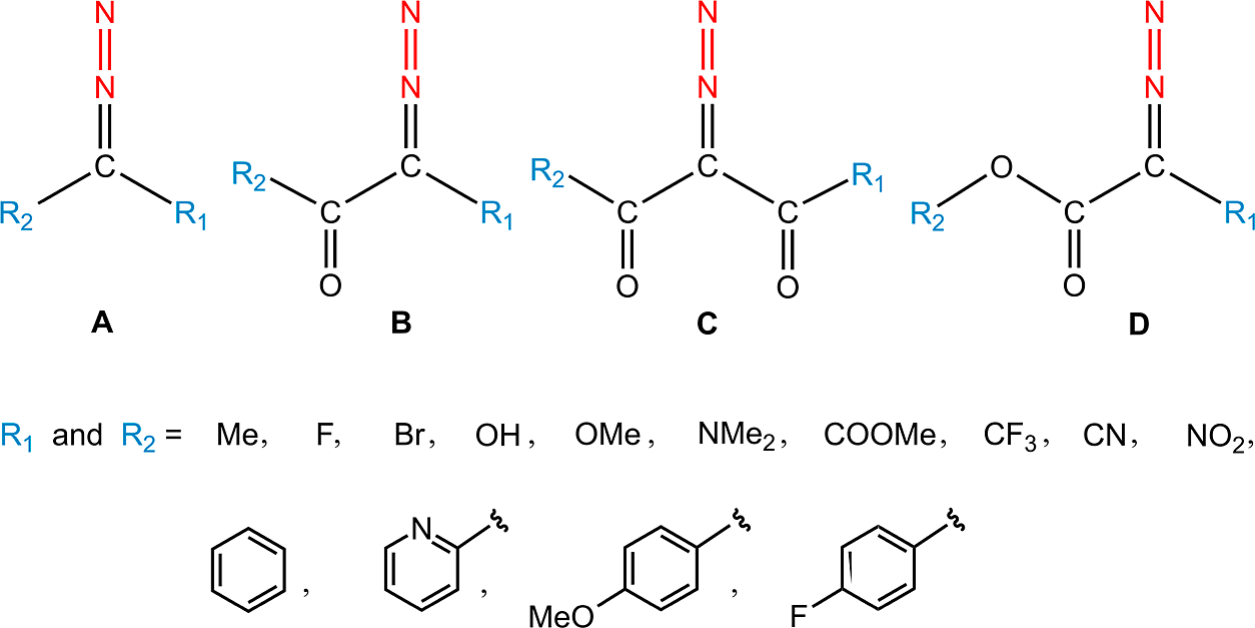
Four Diazo Backbones Accompanied by Different R_1_ and R_2_ Functional Groups to Generate the Database

Theoretical calculations can provide insights into the reactivity of diazo compounds in great detail. In this regard, some density functional theory (DFT) studies have been performed.^31−41^ However, the majority of these studies^31−36,39^ have focused on a subclass of compounds known as α-diazocarbonyl (backbone **B** in Scheme 2) that have been decorated by a limited set of functional groups. For instance, Ariafard et al.^31^ explored the R_2_ substitution impact, whereas the R_1_ site is decorated by a few phenyl derivatives. In this case, the activation barriers ranged from 26.7 to 35.1 kcal/mol. Clearly, the direct attachment of electron-withdrawing or electron-donating functional groups to the carbon carries the diazo group, *i*.*e*., R_1_ site will result in a more pronounced electronic effect and, subsequently, can exert a greater influence on the stability of the transition structure. Moreover, a substantial portion of the studies is devoted to the mechanism underlying the release of N_2_ from diazo compounds in the presence of a catalyst, particularly a metal complex catalyst.^33,37−41^ Taken together, the community still needs a thorough examination of a larger range of diazo compounds to pave the way for a more effective N_2_ gas release process.

The above-simplified description prompted us to conduct a DFT-based investigation, examining a database of diazo compounds consisting of four common backbones decorated with 14 functional groups (Scheme 2). Such a database allows us to discover leading features and optimize them in order to design efficient diazo compounds for N_2_ release. To this end, we also used a machine learning (ML) approach to analyze DFT results and identify relevant chemical descriptors. It is important to note that DFT is an accurate approximation but scales with system size, whereas ML is fast but limited by database size and reliability. By combining these two approaches, we can uncover previously unseen correlations and gain a deeper understanding of chemical reactions.

We aim to address the following questions: (i) how does the structural modification affect the kinetics of the N_2_ gas release? It provides us with information on both the reactivity and stability of the diazo compounds. (ii) Which chemical properties are more associated with the activation energy barrier? It provides us with deeper insights into designing descriptors for high-throughput screening of diazo compounds.

## Results and Discussion

Four common diazo backbones, each decorated with R_1_ and R_2_ substitutions are introduced.^42^ These candidates have received the most experimental attention. The substitutions encompassed a range of electron-donating and electron-withdrawing groups. The combinatorial structure generation leads in duplication when (i) backbones **A**, **B**, and **D** bear −COOMe, −OMe, and −Me as R_2_, respectively, and (ii) backbones **B** and **C** bear –COOMe and –OMe as R_1_, respectively. Then, the corresponding duplicated samples were excluded from the data set. Moreover, sample (F)_2_CN_2_ does not exhibit a stable diazo structure, and the nitrogen molecule is dissociated during geometry optimization. In addition, our efforts to locate the transition structure for five samples in backbone **D**, with R_1_ = –CF_3_ and accompanied by R_2_ = –F, –Br, –CF_3_, –CN, and –NO_2_ were unsuccessful. To this extent, the database consists of 559 samples (**A**: 104, **B**: 182, **C**: 96, and **D**: 177).

**Figure 1:**
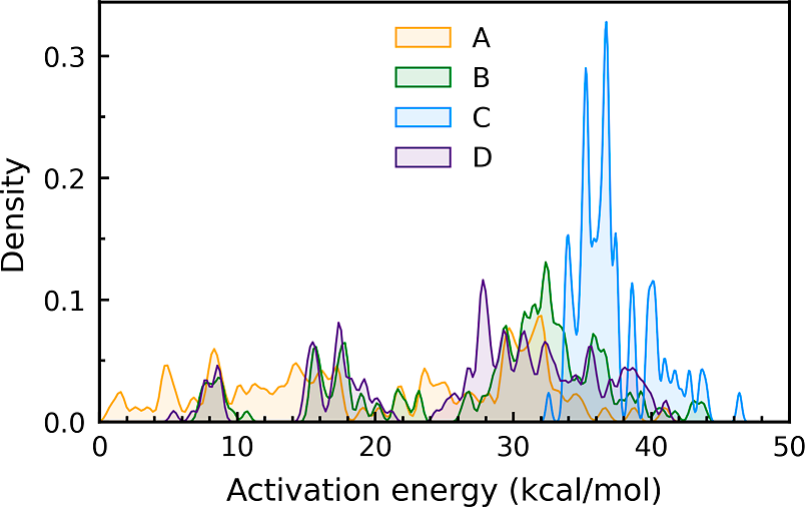
Distribution of the activation energy values for backbones A-D obtained using the SMD/M06-2X/def2-TZVP//SMD/M06-2*X*/6-31G(d) level of theory in dichloromethane for each backbone. The bandwidth of 0.08 smooths the curves.

The activation energy distribution is shown in Figure 1. It demonstrates that backbones **A**, **B**, and **D** are distributed throughout a broad range of 0.8–44 kcal/mol, but backbone **C** is localized at the high activation energy range of 32.5–46.4 kcal/mol. The functional group impact on the activation energy is demonstrated in Figures S1–S3. We discovered that –NMe_2_, –OMe, –OH, and –F, in this sequence, have the greatest impact on lowering the activation energies when directly bonded to the C atom of C=N=N. This effect is amplified when the aforementioned R-groups are simultaneously introduced to both substitution sites. As a result, backbones **A** and **C** have the highest and lowest sensitivity to substitution addition, respectively.

**Figure 2:**
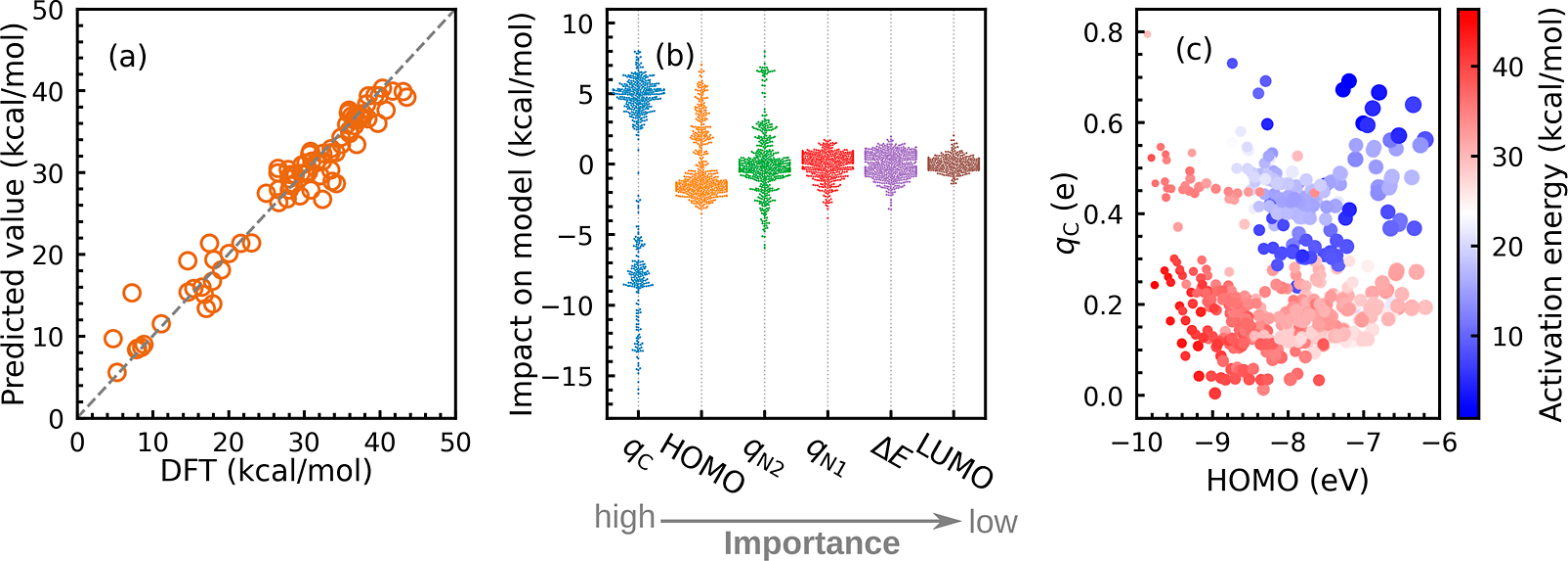
(a) Scatter plots of the DFT values vs predicted values of the trained ML model on attribute-based features. (b) Impact of features on model training. The prominence of features decreases from left to right. (c) Correlation between the activation energy and three most important features, *i*.*e*., HOMO, Mulliken charge on C (*q*_C_) and N2 (*q*_N2_). The marker color shows the magnitude of activation energy [red (high) to blue (low)]. The size of the marker corresponds to absolute *q*_N2_. In our study, N2 always has a negative partial charge; hence, a larger marker size denotes a more negative charge.

To discover the chemical descriptors for rapid evaluation of the transformations, we study the relationship between a list of electronic properties as follows: Mulliken charges of each atom in the C=N=N fragment (labeled as C=N1=N2) as the main moiety of diazo compounds, the highest occupied molecular orbital (HOMO), and the lowest unoccupied molecular orbital (LUMO) energy levels accompanied by HOMO–LUMO energy gaps (Δ*E*) with the target value, i.e., activation energy barrier. Hereafter, the Mulliken charges of the C, N1, and N2 atoms are denoted by *q*_C_, *q*_N1_, and *q*_N2_, respectively. We use the abovementioned properties in feature space to train a random forest^43^ regression model. Figure 2a illustrates the predictability of the trained model. The model achieved *R*^2^ scores of 0.99, 0.91, and 0.91 on training, validation, and out-of-bag sets, respectively. Indeed, a mean absolute error of 1.4 kcal/mol was obtained. This means that the input variables we offer to our ML model are effective enough to detect patterns and learn trends.

In general, sample features can nonlinearly influence activation energy and shift model prediction up or down. Our feature space has *N*_samples_ × *N*_features_ = 3354 variables that all contribute to the predictability. We used the SHapley Additive exPlanations (SHAP) analysis^44^ to decode feature importance and interpret the ML model. The basic principle behind this analysis is to break down a model’s prediction by estimating each feature’s contribution to the prediction based on its missingness. This breakdown indicates how important each feature is in determining the model’s final activation energy forecast. By quantifying the impact of each feature, the SHAP analysis offers a comprehensive and intuitive understanding of the model’s behavior.

The impact of the features on the trained model is shown in Figure 2b. The presence of *q*_C_ causes a positive (up to 9 kcal/mol) or negative (down to −17 kcal/mol) shift in the target value prediction. It has the greatest influence on ML training according to the feature analysis. The majority of HOMOs have a negative influence and are located near 0, but others with a positive impact are spread out to a maximum of 8 kcal/mol. The next notable feature is *q*_N2_, which has tails on both sides but is most frequent at 0. On the model training, the remaining studied characteristics have a minor impact. Figure 2c shows the correlation between the three most important variables and the activation energy. It shows the inter-relationship and gives us crucial insights into how structural and electronic features affect reactivity. According to our findings, the activation barrier energy is lower for compounds that have higher HOMO energy levels, more negative *q*_N2_, and more positive *q*_C_. Backbone **C** is found in the high energy barrier range, where *q*_N2_ is less negative and HOMO energy levels are low. In contrast, other backbones are widely spread.

**Scheme 3:**
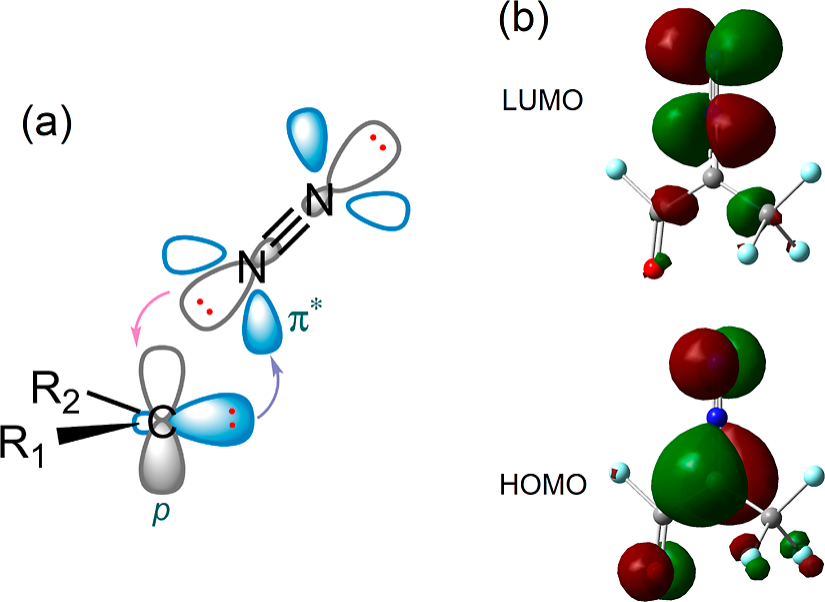
(a) Electron Donation from Nitrogen’s Lone Pair to the Unoccupied Atomic p Orbital of Carbene and π Back-Bonding from Carbon’s Lone Pair to the π* Orbital of N2; (b) Spatial Plots of HOMO and LUMO for a Diazo Compound in Backbone **B**, with R_1_ = F and R_2_ = CF_3_ Balls in cyan, gray, and blue represent H, C, and N atoms, respectively.

Inspired by our ML results, we investigate the relationships further from a chemical standpoint. Carbenes in the singlet state have an sp^2^ hybridized lone pair of electrons and an empty atomic p orbital in their valence shell. The structures of diazo compounds can be explained by sharing the lone pair of the N_2_ dimer with the empty atomic p orbital of the carbene. Conversely, the lone pair of carbenes overlaps with the π* orbital of molecular nitrogen (Scheme 3a). Electron-withdrawing substitutions induce electron deficiency in the carbene carbon, resulting in a weakened electron-donating ability of the carbon and weaker π backbonding of the carbon’s lone pair to the π* of N_2_. As a result, the strength of the connection between carbon and nitrogen weakens and needs less activation energy. We schematized a few compounds in Figures S5–S7 to show how the most crucial characteristics affect the activation energy.

When two orbitals interact, their constructive and destructive interference produces stable and unstable orbitals, and the strength of the interactions determines the energy level of the new orbitals, as well as the energy gap between them. HOMOs in diazo compounds are considerably localized on the carbene fragment, whereas the LUMOs are predominantly located on the diazo moiety (Scheme 3b). Therefore, it can be inferred that the interaction between the carbene and diazo fragments contributes in some way to the formation of HOMO and LUMO in diazo compounds. We expect HOMOs to be an indicator of the strength of bonding between the diazo and carbene components. Accordingly, a diazo molecule with a more stable HOMO requires more activation energy to release N_2_, implying a stronger interaction between N_2_ and carbene components.

**Figure 3:**
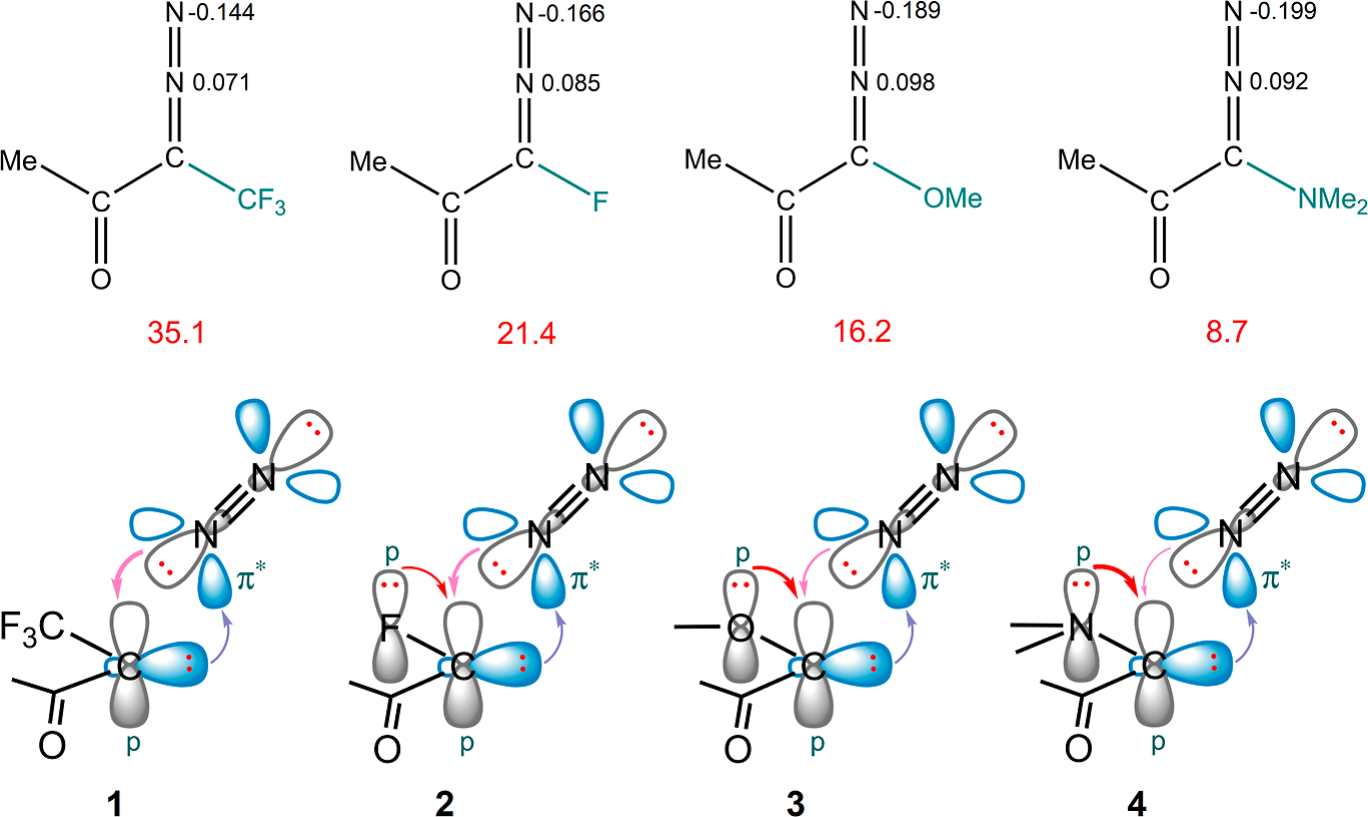
Four compounds of backbone **B** decorated by different R_1_ functional groups while R_2_ is set to the –Me group. The partial charges of N1 and N2 are shown in black, and the activation energy quantities are displayed in red, along with carbene–N_2_ orbital interactions.

More negative partial charges on N2 in diazo compounds result in a lower energy barrier for N_2_ elimination. We choose four chemicals from backbone **B** to make it more understandable (Figure 3). These compounds have –CF_3_, –F, –OMe, and –NMe_2_ as the R_1_ functional group. The ability to donate electrons improves from the first to the last compound.

The lower activation energy for molecule **2**, compared to that for molecule **1**, can be attributed to the presence of fluorine in the vicinity of the diazo group. Although fluorine acts as a strong σ electron-withdrawing group, it can also function as an effective π electron-donor. This competition between fluorine and the diazo moiety to donate electrons to the empty p orbital of carbene weakens the electron transfer from nitrogen atoms. It means that the electron transfer from molecular nitrogen to carbon is reduced, and the electron density remains on the molecular nitrogen. Between the nitrogen atoms, the charge distribution on N1, which is directly bonded to carbon, is more sensitive to the electronic characteristics of the substitutions in diazo molecules. Meanwhile, the next nearest neighbor N, i.e. N2, atom to carbon remains less influenced by structural changes and shows a stronger correlation to the activation energy. Similarly, –OMe and –NMe_2_ groups (molecules **3** and **4**), which are better π-donating groups, cause lower energy barriers for N_2_ release. In contrast, the –CF_3_ group (molecule **1**) which does not have any lone pair on its carbon atom would compete with the electron transfer from the diazo moiety. It is noteworthy that the presence of a methoxy group (−OMe) even on the phenyl ring that is bonded to the carbene carbon leads to a slight decrease in the activation energy. It can be attributed to the increased π electron-donating ability of the 4-methoxyphenyl group compared to the phenyl group and enhances the stability of the transition structure. Figures S4 and S6 contain more examples in this regard.

It should be noted that the electron donation from the nitrogen moiety to the carbene moiety is predominantly manifested in *q*_N2_. However *q*_N1_, which is directly bonded to the carbon atom, is significantly influenced by the attached functional groups. This attribute can effectively reinforce the stability of the transition structures.

**Figure 4:**
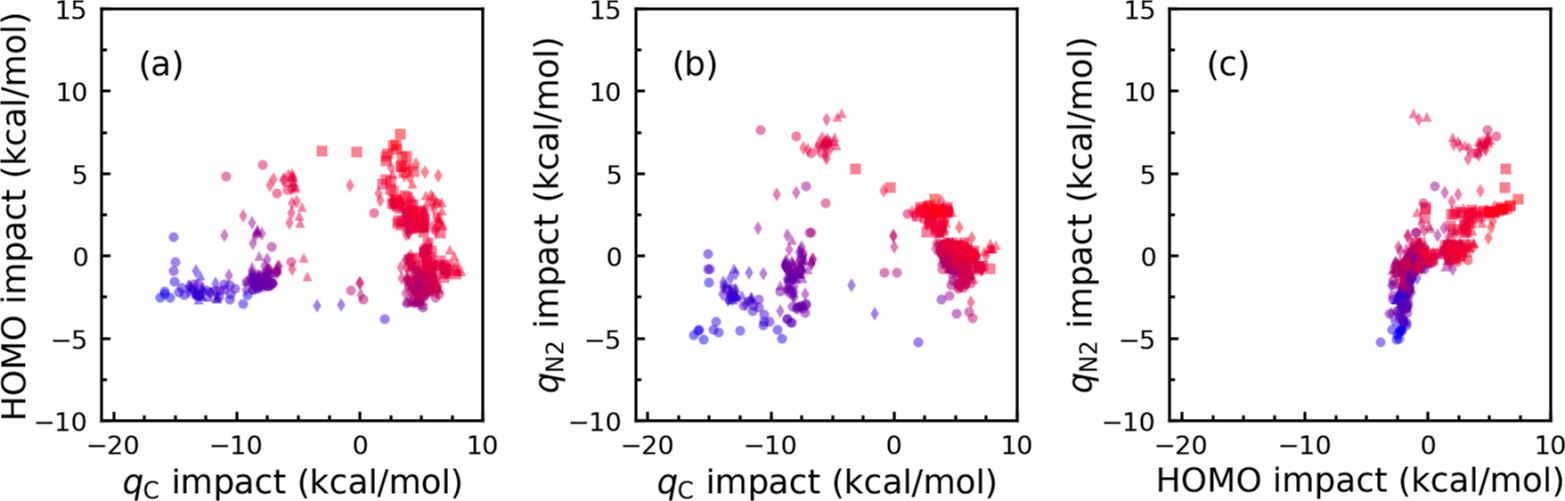
Scatter plots of the impact of (a) *q*_C_ vs HOMO, (b) *q*_C_ vs *q*_N2_, and (c) HOMO vs *q*_N2_ for the model training. The backbones are marked by ○, ◊, □, and △ to indicate **A**, **B**, **C**, and **D**, respectively. The activation energy is represented by marker color; red (high) to blue (low). We formulated color as rgb (red, green, and blue) where *r* = Δ*G*/max(Δ*G*), *g* = 0, and *b* = 1 – *r*. Δ*G* indicates the activation energy barrier.

Figure 4 illustrates the two-dimensional scatter plots corresponding to the SHAP values of the three most important features and activation energies. Our data are clustered into low-, moderate-, and high-range activation barrier energies based on either *q*_C_ vs HOMO or *q*_C_ vs *q*_N2_, while it is hard to detect comparable behavior when using HOMO vs *q*_N2_. It means that *q*_C_ is a decision-maker parameter for model prediction, particularly in the cases of backbones **A**, **B**, and **D**. The low-barrier cluster that localizes at HOMO impact of about 2.5 kcal/mol is dominated by backbones **A** and **B**, the moderate-barrier cluster contains a mix of all except backbone **C**, and the high-barrier cluster is dominated by backbone **C**.

## Conclusions

In conclusion, we have computationally demonstrated the impact of the electronic and structural features on the activation energy required for N_2_ gas release by considering four common backbones of diazo compounds and 14 functional groups. Based on our chemical intuition, we introduced a few attribute-based descriptors. The efficiency of this feature space was examined by training an ML model and explaining its interpretability. Our study resulted in several significant findings, which are detailed below. We conclude that (i) the nature of the functional group which is not directly bonded to the carbon bearing the diazo group has little influence on the activation barrier for N_2_ release, (ii) to lower the activation energy, more positive and negative partial charges on C and N2 are needed, (iii) a more positive C charge decreases the π-donor ability of carbene lone pair to the π* of N_2_ fragment, (iv) the more negative N2 charge shows a weak interaction between the N_2_ lone pair and the carbene vacant p orbital, and (v) the chemical descriptors used for the ML model training led to a powerful predictability that confirms the efficiency of our feature space selection. Our research can contribute to a better understanding of the N_2_ release transformation and gives experimental chemists crucial information about the mechanism of the reactions that is useful in designing new syntheses.

## Computational Details

All DFT calculations were performed using Gaussian 16,^45^ within M06-2X level of theory.^46^ Numerous studies have shown the accuracy of the M06-2X functional in calculating the energy of organic reactions.^47−53^ To fully optimize the geometry of structures and subsequently compute frequencies, the 6-31G(d) basis set^54^ was employed. As most of the experimental studies have been conducted in dichloromethane, we utilized this solvent for the calculations using the SMD implicit solvation model.^55^ Transition structures were located by using the Berny algorithm. To further refine the energies obtained from the SMD/M06-2*X*/6-31G(d) calculations, we carried out single-point energy calculations using a more accurate def2-TZVP basis set.^56^ Tight convergence criteria and ultrafine integral grids were also employed to increase the accuracy of the calculations.

We used the random forest regression ML model, as implemented in the Scikit-learn package of Python.^57^ The optimal set of hyperparameters was computed using a cross-validation score over a grid of predefined space, where the training data were split into 10 groups. The following are the parametrized parameters: “max_depth”: 17, “max_features”: 0.67, “min_samples_leaf”: 1, “min_samples_split”: 2, and “n_estimators”: 50. To generate diazo compound molecule geometries, each backbone is systematically designed by two functional groups using the Maestro modeling interface of Schrödinger material science suite.^58^

## Supporting Information Available

Data distribution; functional group effects; SHAP values; examples of backbones; full source code; data set; Cartesian coordinates, SMILE files, and energies of all calculated species (PDF)
