# Supplementary material for: Kinetics of N2 Release from Diazo Compounds: A Combined Machine Learning-Density Functional Theory Study
Source: ACS Omega. 2023 Dec 15;9(1):1106–12. doi: 10.1021/acsomega.3c07367 (PMC10785077; doi:10.1021/acsomega.3c07367)
Supplement: Supplementary file 1 — ao3c07367_si_001.pdf [file ao3c07367_si_001.pdf]

Kinetics of N_2 Release from Diazo Compounds: A Combined Machine Learning-Density Functional Theory Study

Kaveh Farshadfar[1,*], Arsalan Hashemi[1], Reza Khakpour[1], and Kari Laasonen[1,*]

[1]*Department of chemistry and material science, School of chemical engineering, Aalto University, 02150 Espoo, Finland*

E-mail: Kari.Laasonen@aalto.fi

Contents

1 Data distribution **S3**

2 Functional group effects **S4**

3 SHAP values **S7**

4 Examples of backbones **S8**

1 Data distribution

The activation energy distribution shows that (i) backbone A is distributed over a wide range from 0.8 to 41 kcal/mol with a mean (μ), standard deviation (σ), and median (M) of 20.8, 10.5, and 21.7 kcal/mol; (ii) backbones B and D are similarly distributed from about 5.3 to 43.8, with μ = 28.1, 27.1, σ = 8.9, 8.9, and M = 31.1, 29.2 kcal/mol, respectively; (iii) backbone C is localized at high activation energy values from 32.5 to 46.4, with μ = 37.2, σ = 2.7, and M = 36.7 kcal/mol. The dataset contains 559 samples (compounds).

2 Functional group effects

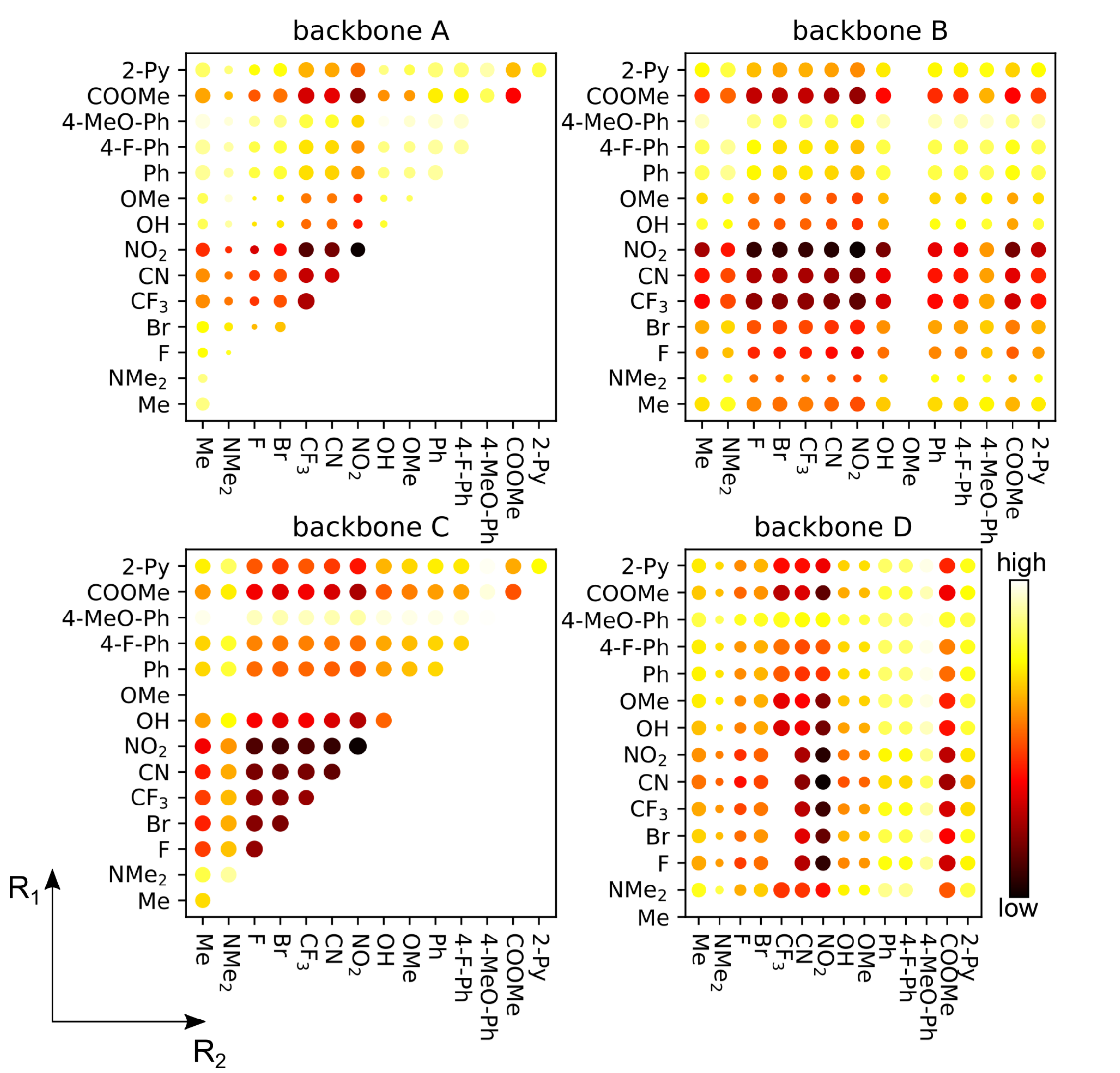

Figure S1: R_1 and R_2 functional groups effect on activation energy in different backbones. The marker's size corresponds to the value of activation energy, and the color gradient corresponds to variations in HOMO energy levels.

The R-groups effect is presented in Figures S1-S3, for each backbone. The (circled) maker size reflects the magnitude of activation energy for N_2 release. The color gradient represents variations in HOMO energy levels, partial charges on C, and partial charges on N2, in Figures S1, S2, and S3, respectively.

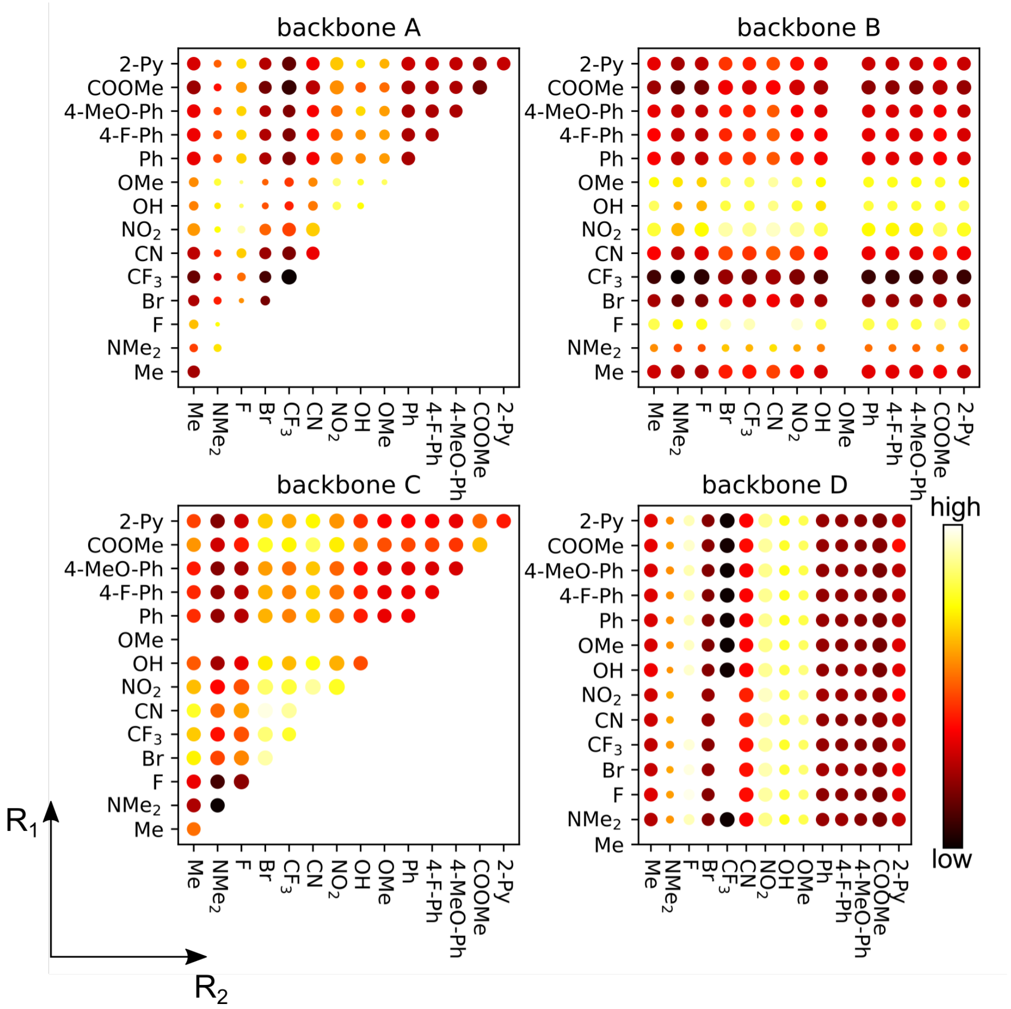

Figure S2: R_1 and R_2 functional groups effect on activation energy in different backbones. The marker's size corresponds to the value of activation energy, and the color gradient corresponds to variations in partial charges on C.

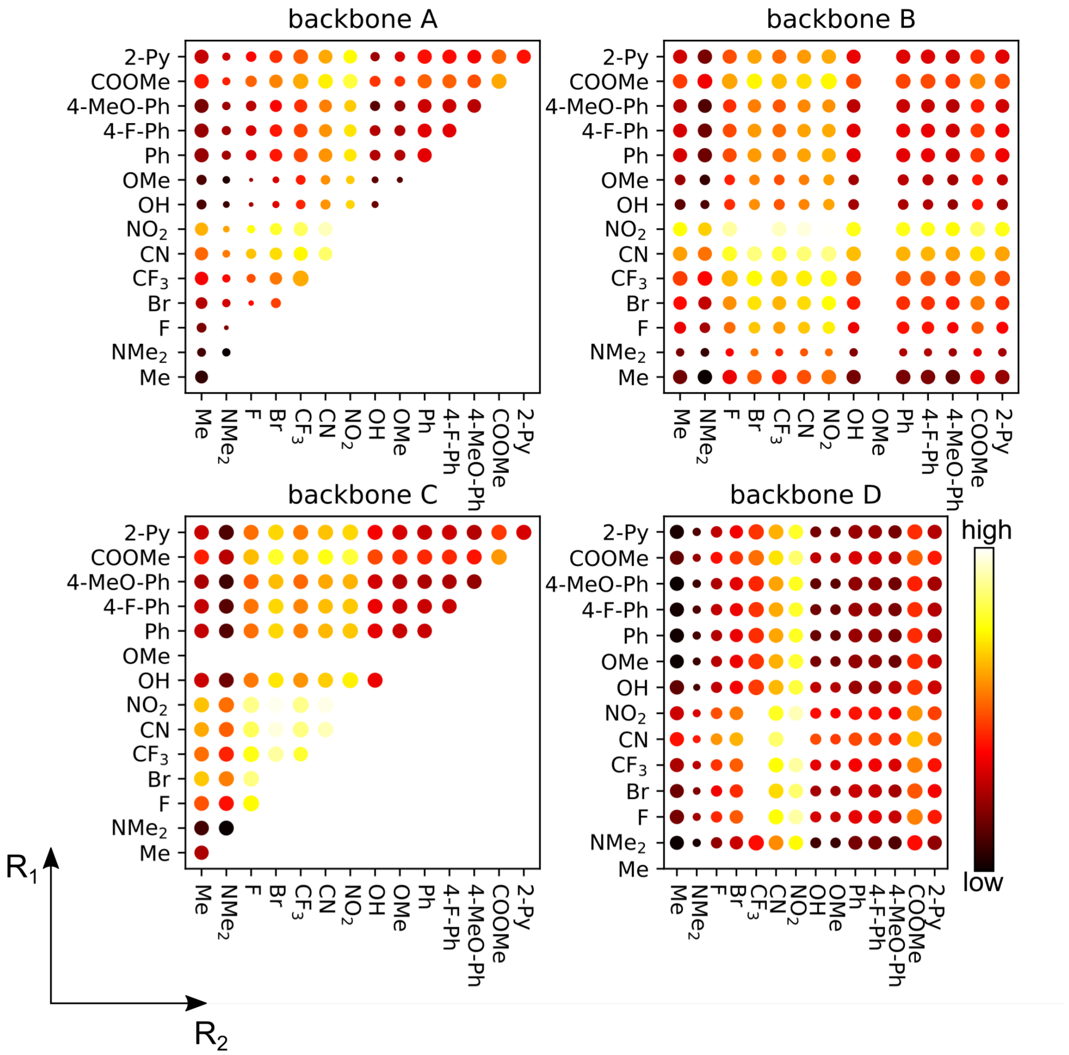

Figure S3: R_1 and R_2 functional groups effect on activation energy in different backbones. The marker's size corresponds to the value of activation energy, and the color gradient corresponds to variations in partial charges on N2.

3 SHAP values

The impact of the features on the trained model is shown in Figure S4. The presence of q_C causes a positive (up to 9 kcal/mol) or negative (down to -17 kcal/mol) shift in the target value prediction. It has the greatest influence on ML training, according to the feature analysis. The majority of HOMOs have a negative influence and are located near 0, but others with a positive impact are spread out to a maximum of 8 kcal/mol. The next notable feature is q_{N2}, which has tails on both sides but is most frequent at 0. On the model training, the remaining studied characteristics have a minor impact.

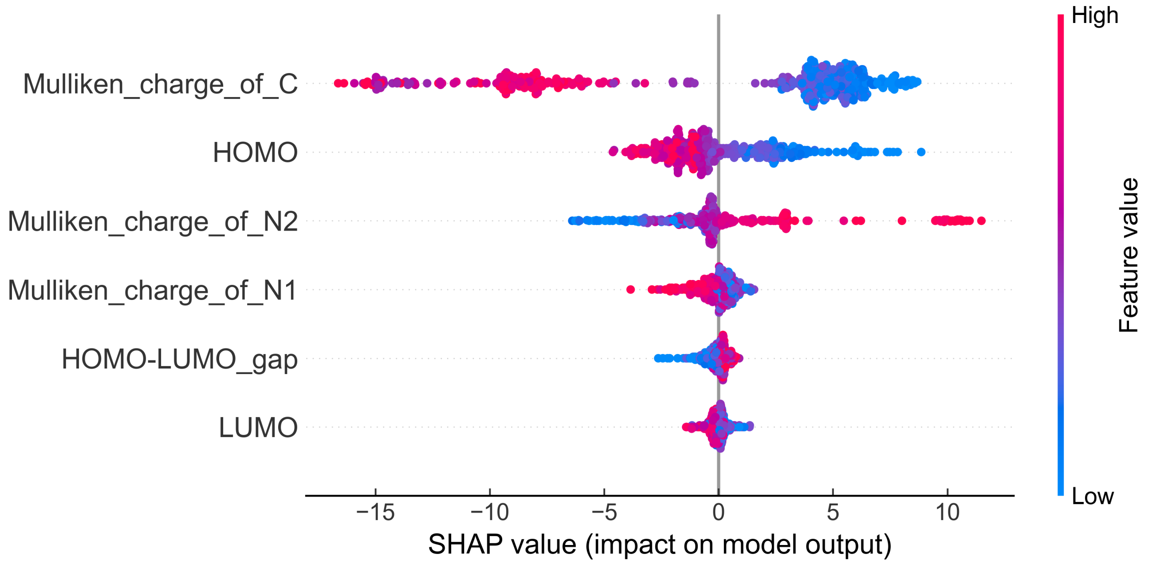

Figure S4: SHAP values for different features in different samples. Colors correspond to the feature values; low (blue) to high (red).

4 Examples of backbones

Figures S5, S6, and S7 show some samples in each backbone with the activation energy required for N_2 release for each sample. Furthermore, for each structure, the three crucial features that significantly affect the magnitude of activation energy, are displayed.

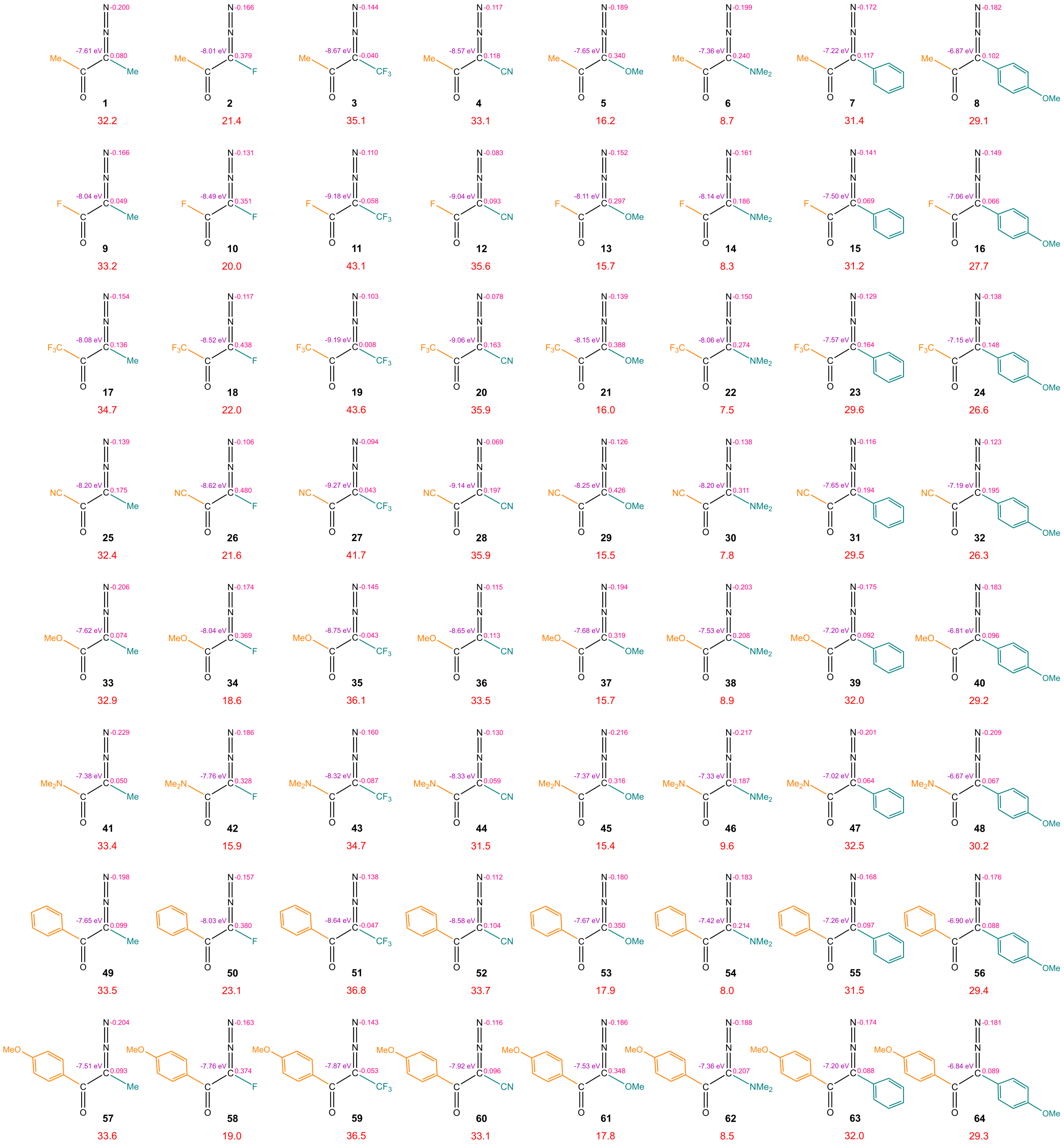

Figure S5: Mulliken charges of the carbon bonded to the diazo group and the terminal nitrogen of the diazo group (in pink), the energy of HOMO (in purple) for a few molecules from the backbone B, and the Gibbs free energy of the corresponding transition state in kcal/mol (in red), obtained from SMD/M06-2X/def2-TZVP//SMD/M06-2X/6-31G(d) calculations, in dichloromethane solvent.

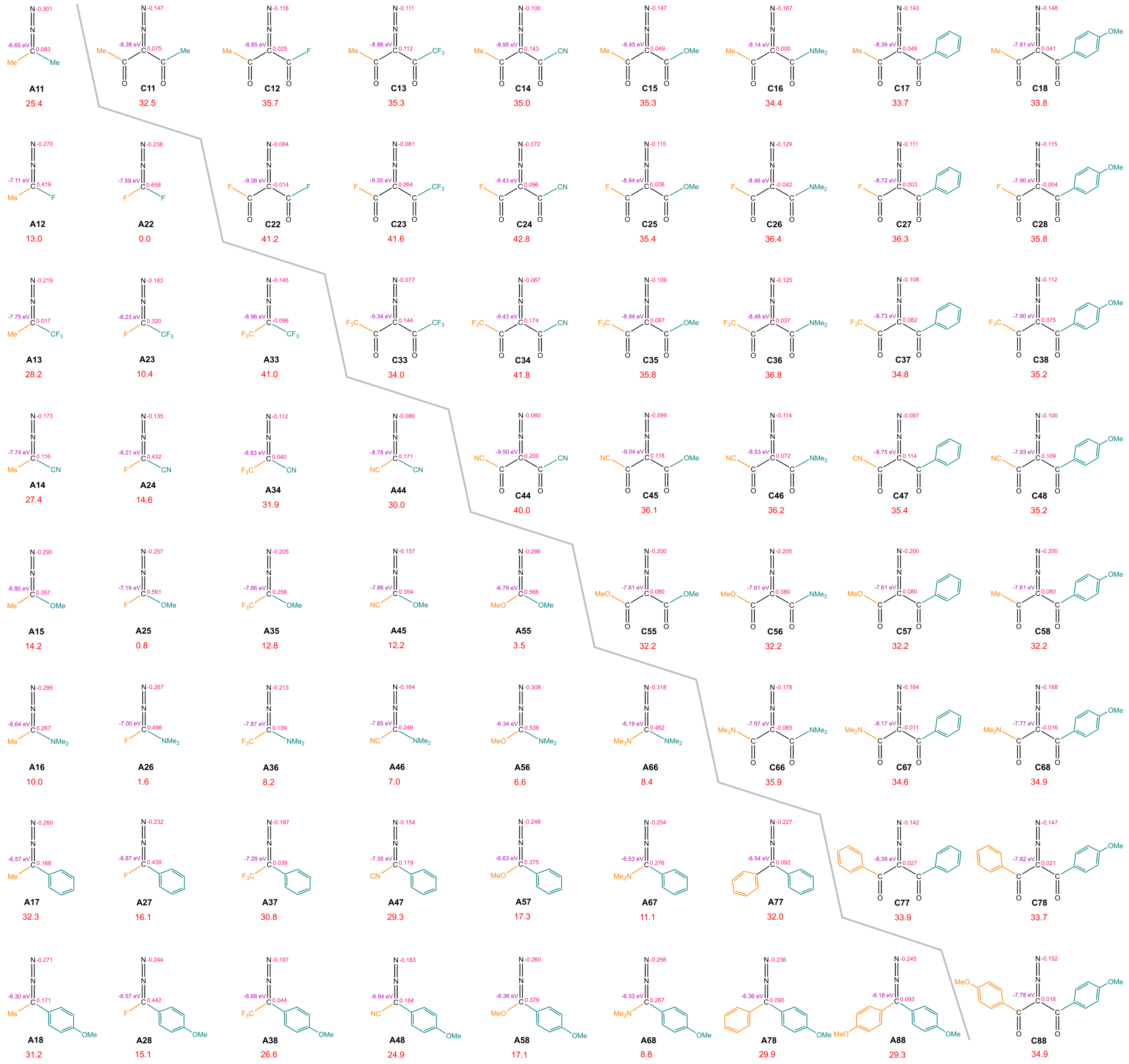

Figure S6: Mulliken charges of the carbon bonded to the diazo group and the terminal nitrogen of the diazo group (in pink), the energy of HOMO (in purple) for a few molecules from the backbones A and C, and the Gibbs free energy of the corresponding transition state in kcal/mol (in red), obtained from SMD/M06-2X/def2-TZVP//SMD/M06-2X/6-31G(d) calculations, in dichloromethane solvent.

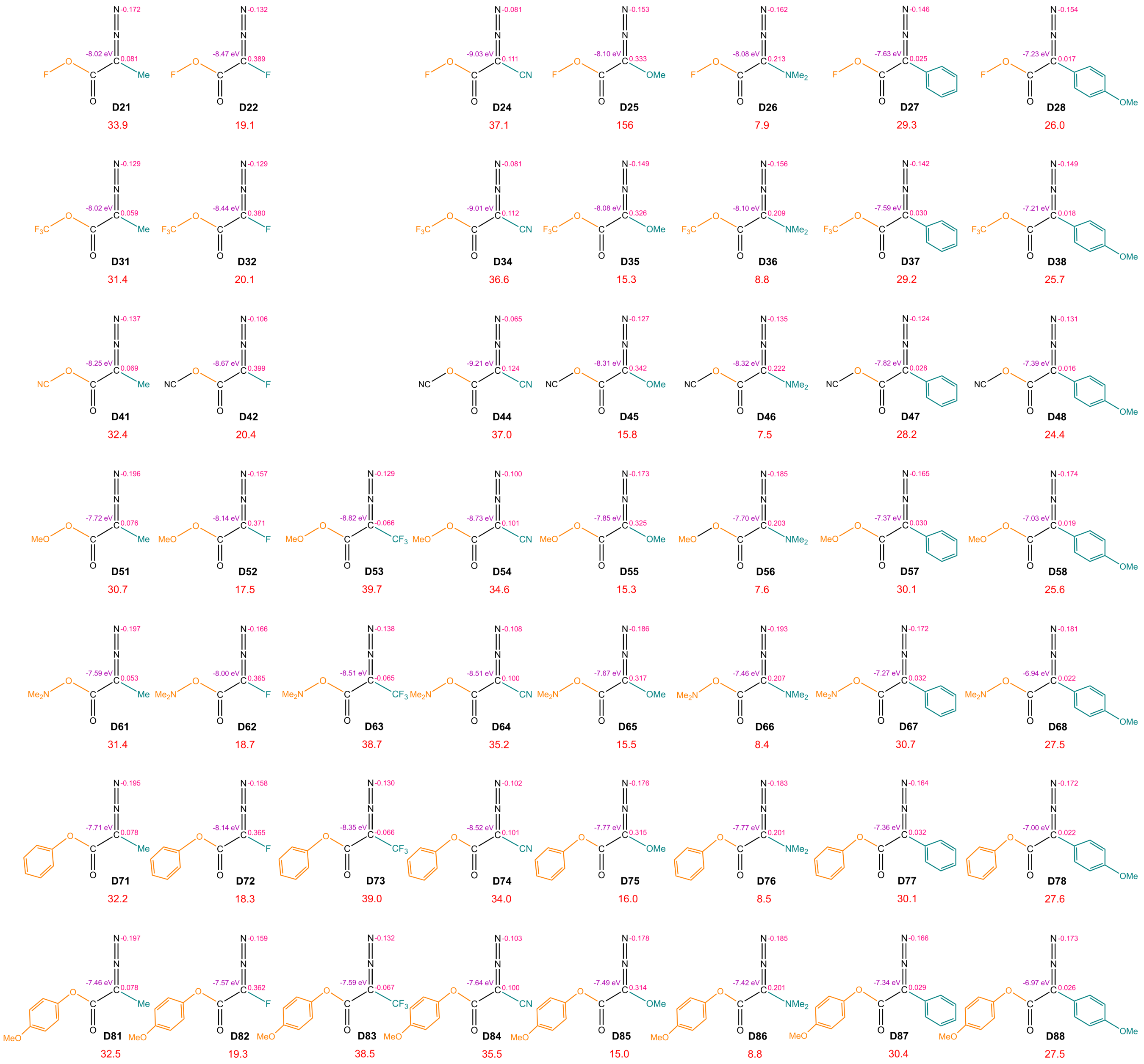

Figure S7: Mulliken charges of the carbon bonded to the diazo group and the terminal nitrogen of the diazo group (in pink), the energy of HOMO (in purple) for a few molecules from the backbone D, and the Gibbs free energy of the corresponding transition state in kcal/mol (in red), obtained from SMD/M06-2X/def2-TZVP//SMD/M06-2X/6-31G(d) calculations, in dichloromethane solvent.
